# Bi-directional interhemispheric inhibition during unimanual sustained contractions

**DOI:** 10.1186/1471-2202-10-31

**Published:** 2009-04-04

**Authors:** Aimee J Nelson, Tasnuva Hoque, Carolyn Gunraj, Zhen Ni, Robert Chen

**Affiliations:** 1 Division of Neurology and Krembil Neuroscience Centre, Toronto Western Research Institute, University of Toronto, Toronto, Canada; 2 Department of Kinesiology, University of Waterloo, Waterloo, Canada

## Abstract

**Background:**

The interaction between homologous muscle representations in the right and left primary motor cortex was studied using a paired-pulse transcranial magnetic stimulation (TMS) protocol known to evoke interhemispheric inhibition (IHI). The timecourse and magnitude of IHI was studied in fifteen healthy right-handed adults at several interstimulus intervals between the conditioning stimulus and test stimulus (6, 8, 10, 12, 30, 40, 50 ms). IHI was studied in the motor dominant to non-dominant direction and vice versa while the right or left hand was at rest, performing isometric contraction of the first dorsal interosseous (FDI) muscle, and isometric contraction of the FDI muscle in the context of holding a pen.

**Results:**

Compared with rest, IHI was reduced at all ISIs during contraction of either type (with or without the context of pen). IHI was reduced bi-directionally without evidence of hemispheric dominance. Further, contraction of the hand contralateral to the conditioning and test pulse yielded similar reductions in IHI.

**Conclusion:**

These data provide evidence for bi-directional reduction of IHI during unimanual contractions. During unimanual, sustained contractions of the hand, the contralateral and ipsilateral motor cortices demonstrate reduced inhibition. The data suggest that unimanual movement decreases inhibition bi-directionally across motor hemispheres and offer one explanation for the observation of ipsilateral M1 activity during hand movements.

## Background

The functional connectivity between the two hemispheres has been studied in cats [1] and monkeys [2,3], and in humans using transcranial magnetic stimulation (TMS). Early work in cats demonstrated both excitatory and inhibitory projections between homologous muscle representations; the territory receiving excitatory input was small in comparison to the large, surrounding inhibited region of cortex that was inevitably stimulated at higher intensities [4]. In humans, interhemispheric interactions between homologous muscle representations may be probed using a paired-pulse TMS technique whereby a conditioning stimulus (CS) is applied to the muscle representation in one hemisphere followed by a test stimulus (TS) to the contralateral hemisphere. The amplitude of the motor evoked potential (MEP) in the muscle contralateral to the TS is suppressed at inter-pulse intervals between 6 and 50 ms [5-7] suggesting that interhemispheric inhibition (IHI) dominates the interaction during this time frame.

Transcallosal connections mediate interhemispheric interactions between homologous muscle representations in the primary motor cortices (M1) [8,9]. IHI is thought to be due to the CS activating an excitatory transcallosal projection that synapses on contralateral local inhibitory interneurons that subsequently inhibit the pyramidal output neurons in the test hemisphere [7,10-12]. In humans, callosal motor fibers are located in the posterior body of the corpus callosum [13-15]. Evidence that IHI is mediated by a transcallosal pathway is derived from patients with callosal abnormalities who did not demonstrate IHI [16-19], the finding that IHI strength increases with the number and density of callosal fibers [20,21] and recording of descending corticospinal volleys that show reduced cortical excitability [22]. Transcallosal projecting neurons are distinct from neurons that give rise to the corticospinal tract [23] and both types are modulated by similar intra-cortical circuits [24]. Within IHI there appears to be a division between a short interval (SIHI) and long interval (LIHI) interhemispheric inhibition. The neurotransmitter and receptor mediating SIHI is not known (14, 17) while LIHI likely involves GABA_B_-mediated inhibition as it has a relatively long time course and is increased by GABA_B _receptor agonist baclofen [25]. The functional significance of the two IHI components remains unknown.

IHI is modulated during muscle contraction. Chen et al., (2003)[26] tested IHI during 50% maximum voluntary contraction (MVC) of the FDI muscle at 8 and 40 ms interstimulus interval (ISI) and found that inhibition is decreased at short ISI (~10 ms). However, it is not known if the timecourse of IHI during contraction will parallel that observed at rest, or whether contraction alters the magnitude of IHI at specific latencies not predicted from the rest state. Also unclear is whether the IHI timecourse will be altered by ispsilateral versus contralateral contractions. This information is important for understanding the role of IHI during unilateral movement where it is predicted that strong IHI will be exerted on homologous muscle representations of the inactive hand. Further, hemispheric dominance may influence the timecourse of IHI during unimanual contraction. Lastly, there remains the issue of whether IHI will be modified by the context of the task [27]. Altering the relevance of the motor task may alter the IHI and provide insight into interactions between motor cortices that underpin purposeful unilateral hand movements.

In the present study, we tested IHI in both cortical directions (right M1 → left M1 and vice versa) in right-handed participants during rest, isometric contraction of the index finger, and in the postural context of holding a pen. There were two goals of the present study. The first goal was to characterize the timecourse of IHI bi-directionally during contraction of either the right or left FDI muscle. We predicted that SIHI will be reduced during contralateral [28] and ipsilateral contraction [29] and that LIHI will reveal a similar modulation. Further, the greatest reduction in IHI during muscle contraction is anticipated when IHI is strongest (i.e. 10 ms for SIHI and 40 ms for LIHI). The second goal was to test the hypothesis that IHI is influenced by the context of the task; IHI will be maximally reduced when the motor task dictates a behaviorally relevant task such as holding a pen and that the release from inhibition will be specific to the right hand, the one that is used for writing in right-handed participants. The present study demonstrates reduced IHI bi-directionally (right M1 → left M1 and vice versa) during unilateral contractions of either hand. These effects are observed for both SIHI and LIHI. IHI was reduced similarly in both isometric contraction and during the context of holding a pen. There was no evidence of hemispheric dominance during rest or any active task condition.

## Results

**Figure 1:**
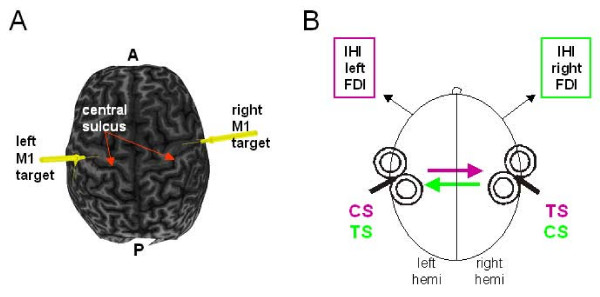
**Experimental set-up**. (A). Illustration of real-time neuro-navigation that provided the location of each coil relative to the FDI target in bilateral M1 for accurate positioning. (B). Interhemispheric inhibition (IHI) was investigated bi-directionally: from the non-dominant to dominant motor cortex (green arrow) and from the dominant to non-dominant motor cortex (purple arrow). IHI is measured in the FDI muscle contralateral to the test stimulus (TS) in each case. The conditioning stimulus (CS) preceded the TS by 6, 8, 10, 12, 30, 40, 50 ms. IHI was tested in both directions during rest, isometric contraction of the FDI muscle (left or right) and isometric pen holding posture (left or right).

A schematic of the experimental set-up is shown in Figure 1. Twelve participants demonstrated both short and long-latency IHI at rest (10 and 40 ms) and were included in further analysis (9 men, mean age 34.8, range 20 – 62 years).

**Figure 2:**
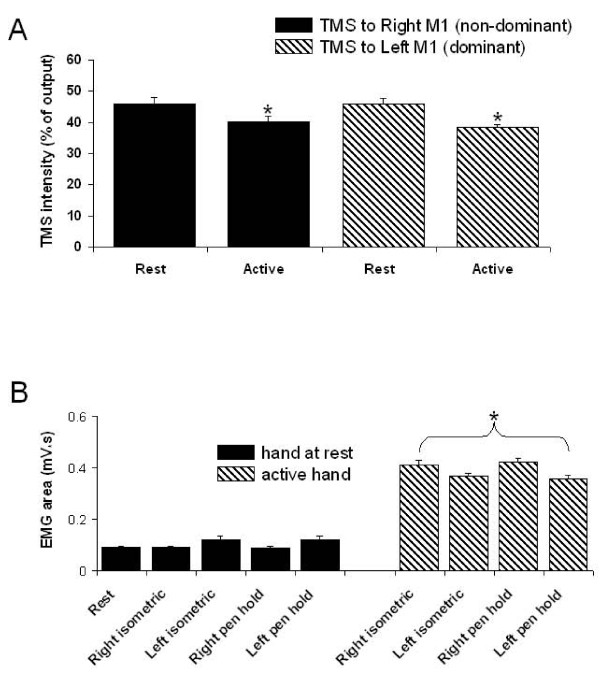
**TMS stimulus intensity and background EMG**. **(A)**. Group averages (with standard error) of TMS stimulator output intensity required to achieve ~1 mV MEP in the contralateral FDI during rest and 20% maximum voluntary contraction of FDI from right and left M1. Stimulus output was significantly lower during active contraction of the contralateral hand. There were no differences in TMS intensity between hemispheres. **(B) **Pre-stimulus EMG area recorded from the left and right FDI muscles while at rest (solid black) and during active tasks (hatched bars). 'Rest' represents the average of both right and left FDI when both muscles were relaxed. There was no difference between conditions while the hand was at rest or when actively engaged. FDI activity was significantly greater while actively engaged compared to resting states. Error bars represent standard error.

The TMS stimulator output intensities used to achieve ~1 mV peak-to-peak MEP for test and conditioning pulses are shown in Figure 2A. The average TMS intensity to achieve 1 mV MEP during contraction of the right and left FDI was 84.8% ± 13.0 (standard deviation) and 87.9% ± 8.5 of the intensity used during the rest conditions. There were no side-to-side differences, demonstrating comparable excitability across the two hemispheres. During contralateral muscle contraction, TMS intensities required to evoke the same MEP amplitude were significantly decreased (F_(3,33) _= 8.41, p = 0.0003) (Figure 2A). Group averaged pre-stimulus EMG area from the right and left FDI muscles are shown in Figure 2B. Solid bars indicate pre-stimulus EMG for the hand at rest and hatched bars indicate EMG during contraction. EMG was significantly greater for the hand performing the task compared to that at rest (F_(8,88) _= 134.0, p < 0.0001).

**Figure 3:**
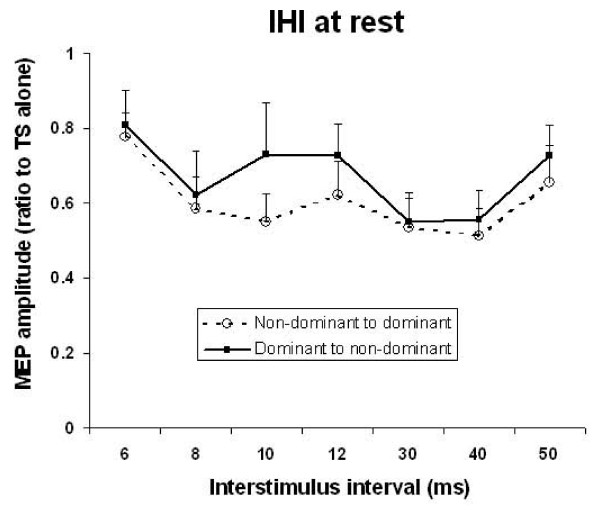
**Resting IHI**. Timecourse of IHI for dominant to non-dominant and non-dominant to dominant hemispheres. MEP amplitude is normalized to MEP amplitude during test stimulus alone. IHI shows similar timecourse and magnitude in both directions. Error bars represent standard error.

**Figure 4:**
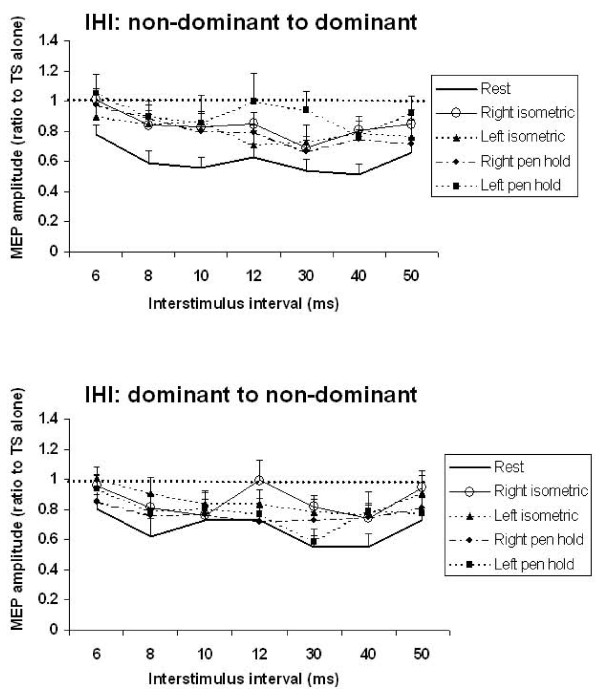
**IHI during all experimental Tasks**. Group averaged timecourse of IHI for each task. The top graph shows IHI recorded in right FDI muscle whereby CS was applied to right M1 (non-dominant) and TS to left M1 (dominant). The bottom graph shows IHI recorded in left FDI muscle. Right isometric refers to isometric contraction made with the right hand while the left hand is at rest, Left isometric is contraction with the left hand while the right hand is at rest, Right pen hold refers to holding a pen with the right hand while the left hand is relaxed, Left pen hold is holding a pen with the left hand while the right hand is relaxed. All active tasks reduced IHI across a wide range of ISIs that included both SIHI and LIHI. There was no difference between the active tasks (isometric versus pen hold) or the direction of IHI (non-dominant to dominant versus dominant to non-dominant). Values above the dashed horizontal line indicate facilitation. Those below the dashed line indicate inhibition.

The three-way ANOVA revealed a main effect of Task (5 levels, F_(4,44) _= 5.17, p = 0.0017), and ISI (7 levels, F_(6,66) _= 5.28, p = 0.0002) but not IHI direction. There were no significant interaction terms. Figure 3 plots resting IHI from the dominant to non-dominant direction and vice versa. The lack of IHI direction effects are clearly observed in the resting state. Post-hoc tests of the main effect of 'Task' revealed that active conditions were not significantly different from each other but were significantly different from rest. Figure 4 shows that the active tasks reduced IHI compared to rest, and isometric contraction with and without pen holding had similar effects on IHI.

Post-hoc testing for the main effect of ISI showed that 8–10 ms and 30–40 ms were significantly different from 6, 12, and 50 ms, thereby revealing the separation of IHI into SIHI (8–10 ms) and LIHI (30–40 ms). To summarize, IHI is similar in depth and timecourse from the right-to-left and left-to-right hemispheres. IHI in both directions reveals two phases of inhibition, the early IHI (SIHI) that is maximal between 8–10 ms and late IHI (LIHI) with maximal inhibition between 30–40 ms.

**Figure 5:**
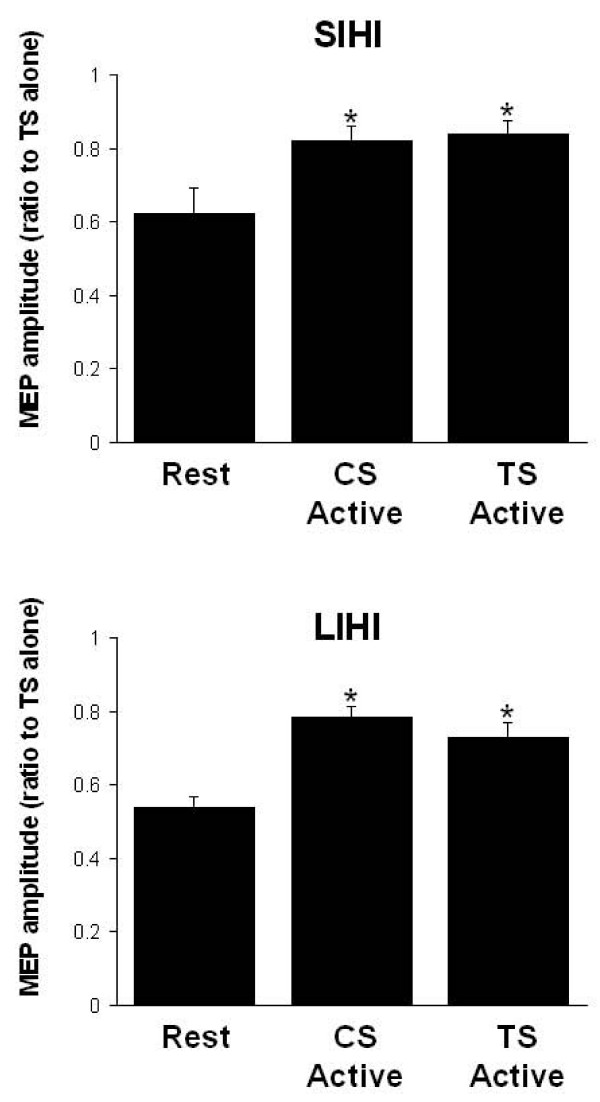
**SIHI and LIHI during rest and contraction contralateral to CS and TS**. Histograms displaying group averaged data (with standard errors) of SIHI (top) and LIHI (bottom). Data combines IHI directions and active tasks (isometric contraction and pen holding). 'CS active' refers to IHI recorded when the FDI muscle contralateral to the CS was contracted. 'Test active' is IHI recorded when the FDI muscle contralateral to the TS was contracted. Both SIH and LIHI demonstrate reduced IHI during active tasks compared to rest. Contraction contralateral to either the CS or the TS reduces IHI similarly. * denotes significant post-hoc tests (p < 0.05) of main effect of Task.

Since our findings are consistent with previous studies showing that SIHI and LIHI are mediated by different mechanisms [30,31], we performed further analysis of SIHI and LIHI separately. To study the effects of ipsilateral versus contralateral contraction on the SIHI and LIHI during rest and contraction, we combined the IHI directions and the context-dependent tasks (isometric and pen holding). For SIHI, a one-way repeated measures ANOVA with factors 'Task' (three levels: rest, conditioning active (hand contralateral to the CS contracting, test active (hand contralateral to the TS contracting)) was performed on the average of ISIs 8 and 10 ms. SIHI analysis revealed the main effect of Task (F_(2,22) _= 6.27, p = 0.0007). Figure 5 (top) plots the tests of the main effect of Task whereby rest demonstrates significantly greater IHI than muscle contraction contralateral to either the CS or TS. However, despite the reduced IHI during active conditions, there remains a net inhibition between the two hemispheres. There was no facilitation observed during sustained contraction. These data indicate that ispsilateral or contralateral muscle activity evokes a global, bi-directional reduction in SIHI. For LIHI, a similar one-way repeated measures ANOVA with factors 'Task' (three levels: rest, conditioning active and test active (hand contralateral to the TS contracting) and data were averaged over ISI of 30 and 40 ms. Figure 5 (bottom) demonstrates the main effect of Task (F_(2,22) _= 13.2, p < 0.0002) and post-hoc tests of the main effects indicate that LIHI is significantly reduced in active conditions compared to rest.

**Figure 6:**
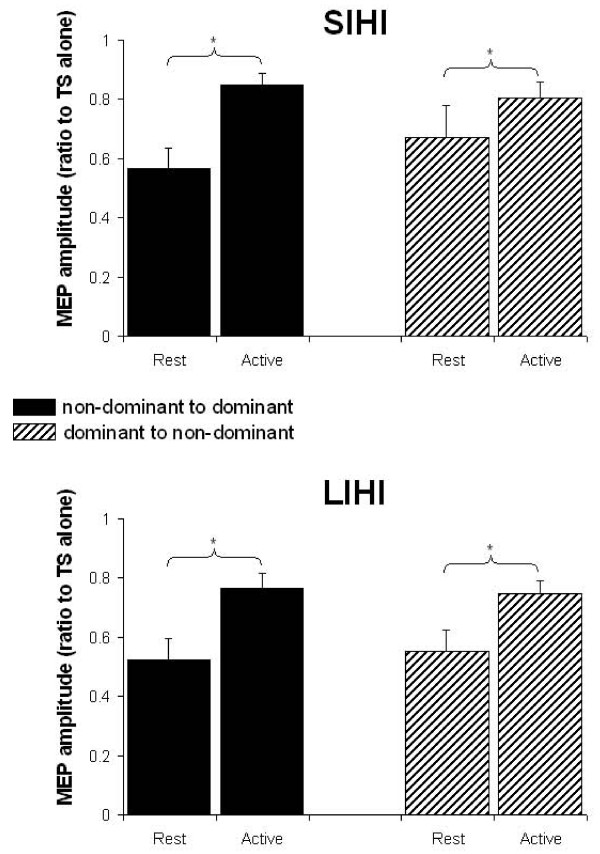
**IHI direction; rest versus all active tasks**. Group averaged data (with standard errors) for SIHI (top) and LIHI (bottom). Both SIHI and LIHI demonstrate reduced IHI during active contraction. The reduced IHI is observed bi-directionally in the right FDI (non-dominant to dominant) and left FDI (dominant to non-dominant). * denotes significant post-hoc tests (p < 0.05) of main effect of Task.

To study the effects of IHI direction and muscle activity on SIHI and LIHI separately, we combined the four active tasks (conditioning or test side active, isometric or pen holding) and compared with rest. For SIHI we averaged data from ISI 8 and 10 ms, for LIHI the data was averaged for 30 and 40 ms ISI. For both SIHI and LIHI, repeated measures ANOVA showed a main effect of Task (rest vs. active) (SIHI, F_(1,11) _= 11.03, p = 0.006; LIHI, F_(1,11) _= 25.75, p = 0.0004) but no significant effect of IHI direction and or Task × IHI direction interaction (Figure 6). Therefore, although there was a trend for greater reduction of IHI with muscle contraction for IHI from non-dominant to dominant hemisphere than IHI from the dominant to non-dominant hemisphere, the differences were not significant. Sphericity was not broken in any of the statistical analyses.

## Discussion and conclusion

We studied the timecourse of bi-directional interhemispheric interactions during rest and tonic contractions of hand muscles in right-handed healthy controls. IHI was reduced bi-directionally during contraction of either hand compared to rest. The reduction occurred across the entire timecourse studied and was similar for the two active tasks – pen hold and simple isometric contraction. Despite the reduced IHI during unimanual contraction, a net inhibitory influence continues to dominate the interhemispheric interaction during tonic contractions. These data indicate that unimanual contractions decrease the amount of IHI bi-directionally, and may provide one explanation for the activity observed in ispsilateral M1 during hand movements.

Immediately preceding the onset a voluntary finger abduction, SIHI is released and changed to facilitation, an effect observed for movements of the dominant hand only [32]. We now extend these findings to show that sustained tonic contraction reduces both SIHI and LIHI bi-directionally (from motor dominant to non-dominant and vice versa). Unlike the effects immediately preceding movement onset [33], sustained contractions did not evoke MEP facilitation but rather reduced the inhibition. Despite the reduction in inhibition, net inhibition was still present. Further, the decrease in IHI was similar for contractions in the right (dominant) and left (non-dominant) hands providing no evidence of hand-dominance effects. Therefore, hemispheric asymmetry of IHI may be present just before a voluntary movement but not during the tonic, sustained contractions tested here.

A previous report found greater SIHI from the motor dominant to non-dominant hemisphere during rest [34]. We observed no such difference in the strength of SIHI, a finding consistent with other reports [35,36]. We now extend this finding to LIHI that was equally strong bi-directionally with no hemispheric dominance during active and rest states. Though SIHI and LIHI may be mediated by different neural interactions, the effects of isometric contraction appear to reduce both similarly, suggesting a common role during simple unimanual contraction.

The postural context of a motor task is capable of modulating intracortical inhibitory circuits [37]. In the present study, we chose to investigate the contextual effects of simply holding a pen in a precision grip at 20% MVC in FDI. The rationale was that performing this task with the right compared to left hand should differentially modulate IHI since this task is strongly under the unilateral control of the right hand. In contrast to our expectation, the pen hold task released IHI similar to the isometric task irrespective of the hand performing the task. One caveat is that the 'pen hold' may have inadequately simulated the physical or strategic context of writing. It is possible that IHI studied during actual writing may reveal context-dependent influences on IHI, and the anticipated dominance effects associated with the right hand. Quantifying the variable forces exerted by individual digits during writing remains a technical challenge though new methodologies may permit future study in this direction [38].

Perez & Cohen (2008)[39] investigated SIHI with the CS positioned contralateral to forearm flexion. Compared to rest, contraction of the arm contralateral to the CS reduced IHI with the greatest reduction occurring at 70% MVC. We tested a similar condition (Task 'B', Table 1) using similar experimental techniques (matching for MEP size) and also observed reduction in SIHI during ipsilateral contraction at 20% MVC. Our data extends these findings to indicate that IHI is reduced in both directions during contraction contralateral or ispsilateral to the CS or TS. To summarize, tonic contraction of a hand muscle in either hand leads to reduced IHI bi-directionally, and this applies to both SIHI and LIHI.

**Table 1:** Task conditions and IHI direction

**Task**	**CS**	**TS**	**IHI recorded**	**Direction of IHi**
A. rest	Left M1	Right M1	Left FDI	D → ND

B. 'iso' right	Left M1	Right M1	Left FDI	D → ND

C. 'iso' left	Left M1	Right M1	Left FDI	D → ND

D. 'pen hold' right	Left M1	Right M1	Left FDI	D → ND

E. 'pen hold' left	Left M1	Right M1	Left FDI	D → ND

F. rest	Right M1	Left M1	Right FDI	ND → D

G. 'iso' left	Right M1	Left M1	Right FDI	ND → D

H. 'iso' right	Right M1	Left M1	Right FDI	ND → D

I. 'pen hold' right	Right M1	Left M1	Right FDI	ND → D

J. 'pen hold' left	Right M1	Left M1	Right FDI	ND → D

One issue encountered in studies of IHI is determining the appropriate CS intensity, particularly when the hand contralateral to CS is active [40]. In the present study, we used a 'matching' technique whereby the CS intensity was set at the output to evoke ~1 mV MEP amplitude when FDI (contralateral to the CS) was active at 20% MVC. In conditions whereby the hand contralateral to the CS was relaxed, CS intensity was adjusted to evoke ~1 mV MEP in the relaxed hand. Thus, we normalized the IHI to changes in corticospinal activity. Using similar techniques, Cohen & Perez report similar findings [41] however when CS intensity was not adjusted for changes in excitability, IHI was not reduced. Since the muscle activity increases MEP amplitude, it is not possible to simultaneously match both stimulus intensities and MEP amplitude for the comparison between active and rest conditions. We choose to adjust the CS intensity to match the degree of corticospinal output because we consider it likely that both the IHI and the corticospinal systems are modulated in a similar manner by voluntary activity. For example, the short interval intracortical inhibition and intracortical facilitation modulates IHI and corticospinal output in a similar manner [42,43]. Therefore, adjusting the CS intensity makes it more likely that the IHI circuits are activated to a similar extent in both rest and active conditions. However, we acknowledge that matching the CS intensity, which would lead to larger MEP evoked by the CS in the active condition, is another option. In our study, the conditioning stimulus intensities used in the active state were about 5% of stimulator output lower than the resting state (Fig 2A). If the lower intensities used in the active state was applied at rest, it will likely result in lower IHI because IHI increases with higher conditioning stimulus intensities ([44] Fig 7A). We observed ~20% difference (expressed as MEP amplitude, ratio to TS alone)(Fig 4 &5) in IHI between rest and active conditions. A previous study [45] showed that a 20% difference in IHI requires a change in conditioning stimulus intensities of about 15% of stimulator output. Therefore, the difference in IHI between rest and active conditions we observed probably cannot be entirely explained by the lower test stimulus intensities used in the active condition, but further studies are needed to address this issue.

The long-held view is that IHI functions to 1) suppress unwanted 'mirror movements' during bimanual movement and 2) to disinhibit motor cortex to produce unilateral contralateral movements [46]. According to these views, it seems counterintuitive that the ipsilateral M1 receives reduced transcallosal inhibition during muscle contraction. This reduced IHI from contralateral to ipsilateral hemisphere may provide one explanation for ipsilateral M1 activity during voluntary contraction of the contralateral limb [47-52]. Suppressive rTMS (1 Hz) to M1 reduces IHI bi-directionally [53] and results in kinematic performance changes in the ipsilateral hand [54]. One possible reason for the shift towards less inhibition bilaterally may relate to the opportunity to efficiently engage either hand during natural movement. Reducing transcallosal inhibition projecting to ipsilateral M1 (ipsilateral to the active hand) may facilitate the rapid engagement of the non-moving hand should the task demand change. Our suggestion is that unimanual hand movements suppress unwanted mirror movements via net inter-hemispheric inhibition but diminish the magnitude of ipsilateral inhibition for efficient engagement of either hand. A testable hypothesis is that IHI magnitude from the contralateral to ipsilateral hemisphere will influence the ability to rapidly engage either hand with greater speed, accuracy or other behavioral gain. The prediction is that IHI strength directed towards the hemisphere controlling the non-moving hand will scale with the ability to engage that hand during contraction of the opposite hand.

## Methods

### Subjects

Fifteen right-handed participants (12 men, mean age 32.9 years, range 20–62 years) were studied. Right-handedness was confirmed at 100% for all participants using a subset of the Oldfield Handedness Inventory [55]. All subjects participated for two three-hour sessions that occurred in a single day and were separated by a lunch break. All participants provided written informed consent in accordance with the Declaration of Helsinki. The University Health Network Research Ethics Board approved the study.

### EMG recording

Surface EMG was recorded from the first dorsal interosseous (FDI) in the right and left hands with 9 mm diameter Ag-AgCl surface electrodes. The active electrode was placed over the muscle belly and the reference electrode over the metacarpophalangeal joint of the index finger. The EMG signals were amplified (1000×), band-pass filtered (2 Hz to 2.5 kHz, Intronix Technologies Corporation Model 2024F, Bolton, Ontario, Canada), digitized at 5 kHz by an analog-to-digital interface (Micro1401, Cambridge Electronics Design, Cambridge, UK) and stored in a computer for off-line analysis. The EMG signal also passed through a leaky integrator and the EMG level was displayed on an oscilloscope (a bright line) to the participant and also transmitted through a speaker for auditory feedback. The position of the bright line was controlled by muscle contraction of either the right or left FDI muscles. Subjects were required to position the line over a second line that marked their level of contraction for 20% MVC. The calculation of 20% MVC was performed prior to TMS. The auditory feedback assisted subjects with maintaining a relaxed muscle state during the rest conditions.

### Neuro-navigation & TMS techniques

Individuals were seated with the chin and forehead rested in Brainsight apparatus (Rogue Research, Canada). Fidicial markers (nasion, tip of nose, left and right interaural notches) were co-registered with contrast markers in the MRI images. MRI was conducted on a 3T GE scanner (172 images) with 3DFSPGR-IR sequences using a 20 cm FOV (256 × 256). TMS was delivered using two custom-built 50 mm diameter figure-of-8 "branding iron" coils (Magstim Company, UK) that were connected to two Magstim 200 stimulators (Magstim Company, Whitland, Dyfed, UK). The branding coil is designed with the handle pointing perpendicular to the plane of the wings of the figure of 8. In this way, the two coils may be positioned nearby without interference from the handles. Each coil was positioned over the scalp area optimal for eliciting a MEP in the contralateral FDI muscle (motor hotspot) with the handle pointing posteriorly at approximately 45 deg to the mid-sagittal line. Each coil was equipped with optical sensors to monitor its position throughout recording. The location and orientation of both coils at the left and right motor hotspots were digitally registered with the MRI using Brainsight Neuronavigation for on-line verification of coil placement and re-positioning between subject breaks. The coils were securely held in position using coil holders mounted on the Brainsight apparatus at either side of the body. Figure 1A displays a typical image using Brainsight software that indicates the location of the motor hotspots within the precentral gyrus for one individual.

The TMS stimulus intensities to evoke ~1 mV MEP in the right and left FDI muscles were determined at rest and also at 20% MVC of right and left FDI muscles. Interhemispheric interactions were investigated by delivering a CS to the FDI motor hotspot in one hemisphere followed by a TS to the FDI hotspot in the opposite hemisphere [7]. In a single 8-minute acquisition, seven CS-TS interstimulus intervals (ISI) and TS alone were presented randomly (6, 8, 10, 12, 30, 40, 50 ms) and each repeated 10 times. Each CS-TS pair occurred once every 5–6 seconds. Five conditions were tested; 1) 'rest' whereby both hands were completely relaxed as determined by on-line EMG recordings 2) Right isometric: isometric contraction of the right FDI at 20% MVC during thumb and index finger press, 3) Left isometric: isometric contraction of the left FDI at 20% MVC during thumb and index finger press, 4) Right pen hold: isometric 20% MVC of the right FDI while holding a pen between the thumb and index finger with the pen tip in contact with the paper, 5) Left pen hold: isometric 20% MVC of the left FDI while holding a pen between the thumb and index finger with the pen tip in contact with the paper. The five conditions were tested while CS was applied to the right M1 and TS applied to the left M1 to test the IHI from the non-dominant to the dominant motor cortex, and while the CS was applied to left M1 and TS to right M1 to test the dominant to non-dominant IHI. The intensity of both the CS and TS was adjusted to evoke a 1 mV MEP in contralateral FDI depending on whether the contralateral muscle was at rest or active. Therefore, for conditions where the FDI muscle contralateral to the TS was at rest, the TMS intensity was adjusted to evoke a 1 mV MEP during rest. For conditions where the FDI muscle contralateral to the TS was performing 20% MVC, the TMS intensity was adjusted to evoked a 1 mV MEP in the active muscle. This is an important consideration since the degree of IHI is dependent on the intensity of the CS and TS [7]. Trials contaminated by EMG in the resting hand were rejected on and off-line. Rest motor threshold was not measured in this study. Table 1 outlines the task conditions and the direction of IHI tested.

### Data Analyses

The peak-to-peak MEP amplitude was measured offline. The paired-pulse MEP amplitude was expressed as a ratio of the mean unconditioned MEP amplitude (TS alone) for each participant. Ratios below one represent inhibition and ratios above one represent facilitation. The area of pre-stimulus EMG was calculated for a 18 ms window prior to the first TMS pulse for each trial in each condition. This measure was used to examine background EMG across isometric contraction tasks and also to calculate the background EMG at rest.

Three-way repeated measures ANOVA was performed with independent factors *IHI direction *(2 levels; non-dominant to dominant, dominant to non-dominant), *task *(5 levels; rest, Right isometric, Left isometric, Right pen hold, Left pen hold), and *CS-TS interval 'ISI' *(7 levels; 6, 8, 10, 12, 30, 40, 50 ms). Pre-stimulus EMG and TMS output intensity were each tested separately using one-way ANOVA with independent factor '*task*'. Sphericity was assessed using the Mauchly's criterion. To assess changes in IHI during active tasks, only participants demonstrating short (10 ms) and long-latency (40 ms) IHI at rest with at least 10% reduction in MEP amplitude were included in statistical analyses.

## Abbreviations

(IHI): Interhemispheric inhibition; (LIHI): long interval interhemispheric inhibition; (FDI): first dorsal interosseous; (SIHI): short interval interhemispheric inhibition; (TMS): transcranial magnetic stimulation; (TMS): transcranial magnetic stimulation; (MEP): motor evoked potential.

## Authors' contributions

AN conceived the study, carried out data collection, data analysis, and writing the manuscript. TH carried out data collection and data analysis, edited the manuscript. CG conceived the study, carried out data collection, edited the manuscript. NZ conceived the study, edited the manuscript. RC conceived the study, assisted with data analysis, provided substantial edits to manuscript. All authors read and approved the final manuscript.
